# The Relationship between Income and Morbidity—Longitudinal Findings from the German Ageing Survey

**DOI:** 10.3390/ijerph182312365

**Published:** 2021-11-24

**Authors:** Elena Reche, Hans-Helmut König, André Hajek

**Affiliations:** Department of Health Economics and Health Services Research, University Medical Center Hamburg-Eppendorf, Martinistr. 52, 20246 Hamburg, Germany; h.koenig@uke.de (H.-H.K.); a.hajek@uke.de (A.H.)

**Keywords:** income, chronic conditions, morbidity, health

## Abstract

It is often assumed that higher income contributes to physical health. Indeed, there is a huge amount of research showing a strong significant association between income and health. However, very few studies have used longitudinal data and an objective variable for health, such as morbidity. Therefore, this study aims to examine the association between the income and morbidity of individuals over time. Data from a total of four waves (year 2008 to year 2017) of the nationally representative German Ageing Survey was analyzed by linear fixed-effects regressions. The used equivalized income was based on the respondents’ monthly net household income. To obtain a comprehensive picture of the dependent variable morbidity, self-reported diseases, current symptoms, and physician-diagnosed diseases were examined. The analyses showed no significant association between percentage changes in income and morbidity in the total sample. Even after considering selected socioeconomic groups in further subgroup analyses, there was no significant within-person association found over time. In summary, the unexpected results of this study suggest that the previously assumed link between income and health in Germany may be called into question. Further research based on longitudinal studies is, therefore, required.

## 1. Introduction

For most people, it seems obvious that income and health are strongly linked. They would expect health to be better the more income someone has. To be more precise, it is often assumed that healthy lifestyle choices, such as nutrition and exercise, are easier to access with higher income. In fact, most are probably aware that healthy foods such as fruits and vegetables are more expensive than junk food [1]. The opportunities for physical activity in the form of sports clubs and gyms are also likely to depend on the amount of money available [2]. Another explanation for this assumption could be the use of additional services at the doctor’s office in Germany, which are not paid by health insurance funds and, thus, may also depend on individual income [3].

In terms of general health, studies have shown that there is a positive association between income and health by using both cross-sectional [4,5] and longitudinal data [6]. However, some factors that reduce the robustness of these previous findings should be considered. While there are a great number of cross-sectional studies on this topic, significantly fewer studies used longitudinal data [7]. The positive association between income and health turned out to be clearly smaller in longitudinal studies than in cross-sectional studies [8], and some longitudinal studies even found a significant negative relationship [9]. In addition, the vast majority of studies examined self-rated health, often quantified using a single item. Although they are, in principle, subjective, physical as well as mental health are summarized in the measure of self-rated health, which remains an easily biased variable due to the subjective evaluations of each respondent. Since self-rated health is a subjective measure, it is also relevant to consider a more objective variable in this context, such as morbidity. Previous studies have found a significant negative or even nearly linear relationship between income and different measures of morbidity. To put it more precisely, morbidity decreased as income increased [10,11,12]. However, all of them used only cross-sectional data. In contrast, a three-year prospective follow-up study from China showed that there was no significant association between income and morbidity [13]. Nevertheless, to date, there have been very few studies investigating the link between income and morbidity based on longitudinal data [14].

In view of the fact that longitudinal data has more explanatory power than cross-sectional data [15], there is a research gap in the literature. Therefore, the aim of this study is to investigate the association between income and morbidity using longitudinal data.

By focusing on the older population aged 40 and above, our study particularly considers the age structure prevailing in industrialized nations caused by demographic change. In Germany, for example, 31% of the population can be expected to be older than 65 years by 2060 (DZA 2017). For this reason, it is of particular interest to investigate the association between income and morbidity in older adults. In addition, it is well known that as age increases, so does morbidity, and, therefore, a possible relationship may appear more evident in this age group.

In the following analyses, morbidity was examined in different ways to provide a more comprehensive view of the association between income and morbidity. We investigated the association between percentage changes in income and patients’ self-reported information on the (i) number of chronic conditions as well as their (ii) severity. Moreover, the relationship between income changes and (iii) the number of physician-diagnosed diseases was examined.

We hypothesize that an increase in income is associated with a decrease in morbidity. As mentioned above, we assumed that morbidity prevention, such as healthy nutrition, frequent physical activity, and good quality of received health care, could be more easily realized with higher income [16]. Furthermore, our hypothesis is in accordance with previous research (e.g., [17,18]).

## 2. Methods

### 2.1. Sample

The data analyzed in this study originated from the German Ageing Survey (DEAS), to which the German Center of Gerontology (DZA) provided access. The DEAS is funded by the Federal Ministry for Family Affairs, Senior Citizens, Women and Youth (BMFSFJ). The survey started with its first wave in 1996 and continued to interview only community-dwelling adults at regular intervals in 2002, 2008, 2011, 2014, and, most recently, in 2017. It used a cross-sectional sample as a baseline sample in 1996 and combined re-surveyed baseline samples with panel samples in the following waves.

Based on the long observation period of more than two decades, the German Ageing Survey meaningfully represents the German population in the second half of life. For this purpose, more than 20,000 people aged 40 years and older were, so far, surveyed in interviews on a wide range of topics. Using a two-stage sampling procedure, a random sample of 290 municipalities was drawn from a total of 12,000 municipalities in Germany in the first survey year (1996). The subsequent basic samples were created based on the local civil registers of these 290 municipalities. Participant selection was based on a sample stratified by age, gender, and region. The preconditions for participation in the panel survey were a written consent of the individual to participate in the panel and the existence of at least one interview in previous waves. Participants also had to be still alive and not living abroad. The participants answered questions about demography, household composition, employment status and economic situation, health, psychological resources, marriage, and family as well as social networks, leisure activities, the view on aging, and critical life events. The variable of physician-diagnosed diseases was first surveyed in 2008 (Wave 3). Thus, this study analyzed data only from Wave 3 onwards. All variables included in this study (dependent variables, independent variables, and potential confounders) were included in 2008, 2011, 2014, and 2017. Within this period, the survey acquired four panel samples and two additional baseline samples. The response rate of the panel samples increased steadily from 2008 to 2017, from an initial 54% in 2008 to 58% (2011) and 61% (2014) and finally 63% in 2017. This response rate is comparable to other large German surveys [19]. In contrast, the response rate of the baseline sample dropped from 39% (2008) to 25% (2014), reflecting the trend of declining participation rates in initial participation of survey studies in Germany. The methods of this survey did not involve invasive procedures or other risks for the participants, so the approval of an ethics committee was not necessary. In addition, all participants provided written informed consent. The study was also conducted in accordance with the Declaration of Helsinki (and its later amendments).

### 2.2. Dependent Variables

To analyze the relationship between income and morbidity as widely as possible, we applied three different morbidity variables in this study. At first, a list of the 11 most common diseases was used to measure the number of self-reported diseases. The list contains cardiovascular problems, bad circulation, joint/bone/back problems, respiratory problems, intestinal problems, cancer, diabetes, liver/kidney disorders, bladder problems, and eye and ear problems (see Table S1 in the Supplementary Materials for a detailed list). The Charlson Comorbidity Index, which is widely used to classify comorbidity and predict the risk for mortality, contains a very similar list of diseases [20]. In our survey, a scale from 1 (none) to 4 (severe) for current complaints reflected the severity of the aforementioned chronic illnesses and was also self-reported via a questionnaire.

To further assess morbidity, the variable of physician-diagnosed diseases was also used. For this purpose, a list of 19 common diseases has been developed; this list is more specific about some diseases than the abovementioned list of self-reported diseases. For example, cardiovascular diseases are classified as high cholesterol, high blood pressure, heart attack, heart failure, and stroke. Poor circulation is indicated separately for the brain and the leg. Furthermore, the categories osteoporosis, arthritis, and rheumatism are available in addition to joint complaints. The list has also been supplemented with additional conditions such as incontinence, mental illness, and Parkinson’s disease. On the other hand, cancer and respiratory diseases were included in the list without specifics. Gastric ulcer was reported as the most common proxy of intestinal problems, as were glaucoma and macular degeneration as proxies for eye problems (see Table S1 in the Supplementary Materials for a detailed list). The list described was developed with a combination of the Charlson Comorbidity Index [20] and Functional Comorbidity Index [21], as well as consultations with geriatric specialists. There are other large surveys, such as SHARE, that use a list of common chronic diseases nearly consistent with the list above [22]. In addition, physician-diagnosed diseases are surveyed in a very similar form in both the German Health Interview and Examination Survey for Adults (DEGS) [23] and the German Health Update (GEDA) [24]. Analyzing the association between income and morbidity, we did not consider individual diseases but instead formed respective count scores. The used count scores are superior to single conditions because, for example, possible accordance between self-reports and medical reports is easier to verify. In addition, the results can be presented more clearly, thanks to the simplification [25,26].

### 2.3. Independent Variables

The survey measures income by asking individuals about their total monthly net household income in euros. This income includes wages, salaries, self-employment income, and any form of retirement benefits. In addition, all forms of public aid and child and housing allowances are taken into account. Income from rents, leases, and interest, as well as sickness benefits and long-term-care insurance benefits, are also included in net household income. Finally, the total monthly net income of all persons living and running their households together is summed up. The data was not taken from an income register but was self-reported.

It is also worth mentioning that income generally refers to regular cash flows such as wages, pensions, or interests. In contrast, assets include individual possessions such as traditional savings, real estate, or even shares and material values. This study considers only household income or, more precisely, the percentage change in household income.

Equivalized income provides better comparability of living standards in different households by considering household size and its composition [27]. For this reason, the net equivalent household income was mainly used as the independent variable in this study. Income was weighted according to the modified OECD equivalent scale used by Eurostat and the German Federal Statistical Office [28,29]. The net equivalent household income has also been used by other large surveys such as the German Socio-Economic Panel (GSOEP) [8]. For the analyses, we additionally logarithmized the net equivalent household income to approximately represent the percentage change in income. This provides better comparability between different income groups. In order to check the robustness of the analyses, logarithmized net household income was also used in this study (see Table S2 in the Supplementary Materials for an overview of the used variables).

### 2.4. Potential Confounders

Based on theoretical considerations and empirical studies [7,30,31], covariates were selected. We included sociodemographic, psychosocial, and health-related covariates in the regression analyses. As sociodemographic factors, we included sex, age in years, education level (based on the International Standard Classification of Education (ISCED-97) [32] and summed up in three groups), family structure (married, living together with spouse; married, separated from spouse; widowed; divorced; single), employment status (working, retired, other: not working), and social class (stratified in five groups [33,34]). These factors are important to consider since previous studies have shown that marital status is associated with health. For example, it was found that married people are healthier than unmarried people [30]. Moreover, it has been shown that being married is associated with higher income [35]. It is also known that higher levels of education can contribute to better health [36]. Furthermore, higher education is associated with higher income [37]. Moreover, psychological factors such as social network (number of important people in regular contact), loneliness, and life satisfaction were also considered. Loneliness was quantified using the 6-item De Jong Gierveld Loneliness Scale [38], and life satisfaction was surveyed with the help of the Satisfaction With Life Scale (SWLS, from 1 to 5, with higher values reflecting higher life satisfaction) [39]. Regarding these psychosocial factors, studies have been able to show that higher life satisfaction is associated with both higher income and lower morbidity [31]. The health-related covariates include self-rated health (ranging from 1 = very good to 5 = very bad), depressive symptoms, and physical functioning. To measure depressive symptoms, the 15-item Center of Epidemiological Studies depression scale (CES-D) was used, ranging from 0 to 45, with higher values indicating higher depressive symptoms [40,41]. Physical functioning was assessed by the subscale “Physical functioning” of the Short-Form Health Survey (SF-36), ranging from 0 (worst) to 100 (best) [42]. In this context, it has been shown that higher income improves self-rated health significantly [6]. In addition, studies have found that lower income and a higher number of chronic conditions are related to a higher prevalence of depressive symptoms [43]. It should be noted that we used the changes in time-varying covariates over time in fixed-effects regression analyses (please see the next section for further details).

### 2.5. Statistical Analysis

We used linear fixed-effects regressions to examine the association between income change and morbidity. To investigate this association, we considered the abovementioned possible confounding and influencing factors. In this context, time-constant and time-variable factors can be distinguished. By using fixed-effects regressions, all time-invariant differences between individuals, such as sex or personal characteristics, are controlled and only intra-individual changes over time are identified. In contrast to fixed-effects regressions, random-effects regressions result in inaccurate estimates if unobserved time-constant factors are associated with outcome measures [44]. In this case, fixed-effects regressions show consistent estimates (when the exogeneity assumption holds), and, therefore, they were used in our study.

Additional exploratory analyses were conducted, stratified by age (two groups: 40–64 years and 65 and older), sex (two groups: women and men), education level (three groups: low, medium, high), and income groups (two groups formed by a median split: low and high), to determine whether the relationship between income change and change in morbidity was specific for any of these socioeconomic groups. Thus, it should be emphasized that using FE regressions, we examined whether changes in income are associated with changes in morbidity. It is also worth noting that a standard set of potential confounders were used, irrespective of how morbidity was quantified in regression models. To address the problem of heteroskedasticity occurring in the regression analyses, we used robust standard errors in the study. For statistical significance, *p* < 0.05 was used. The statistical analyses were performed using the program Stata/MP 16.1 (Stata Corp, College Station, TX, USA).

## 3. Results

### 3.1. Sample Description

A total of 13,027 observations with an average age of 62.4 years (±10.8 years) were used. The gender distribution, with 49.7% women and 50.3% men, was almost symmetrical.

The mean net household equivalent income was EUR 1847.2 (±1497.2; ranging from EUR 1 to 65,000), and there was an average net household income of EUR 2859.1 (±2317.7; ranging from EUR 1 to 97,500). Furthermore, individuals self-reported an average of 2.4 (±1.8; ranging 0–11) chronic conditions, which is nearly equal to the average of physician-diagnosed diseases of 2.3 (±1.7; ranging from 0 to 14). Finally, individuals reported a mean severity of their illnesses of 6.6 (±5.7; ranging from 0 to 41). See Table 1 for more details.

### 3.2. Regression Analysis

The results of the fixed-effects regression analyses are shown in Table 2 (coefficients of the confounders are shown in Table S3 in the Supplementary Materials). The different sample sizes can be explained by small differences in missing data on outcomes. In all regression models presented, we adjusted for the same set of potential confounders.

After controlling for several potential confounders, the fixed-effects regression analyses found no significant relationship between change in log income and self-reported diseases (ß = −0.03, *p* = 0.64). Additionally, for a robustness check, the log net household equivalent income was replaced by the log net household income. However, the results remained insignificant (ß = −0.05, *p* = 0.43).

When considering the morbidity variable ‘physician-diagnosed diseases’ again, no significant relationship with change in log income was found (ß = −0.04, *p* = 0.41). Replacing log net household equivalent income with log net household income did not significantly change the result. A correlation could still not be shown (ß = −0.03, *p* = 0.56).

Similarly, when the morbidity variable ‘self-reported severity of diseases’ was examined, no significant relationship with log income change was found (ß = −0.16, *p* = 0.37). Using the same robustness check as above, which is replacing log net household equivalent income with log net household income, we found that the results did not change either. Thus, the results remained non-significant here as well (ß =−0.11, *p* = 0.57). For further information, see Table 2.

We also checked whether the association between log income and morbidity remained insignificant when we only adjusted for (i) sociodemographic factors (see Table S4 in the Supplementary Materials) or (ii) sociodemographic and psychosocial factors (see Table S5 in the Supplementary Materials).

Again, the associations of interest remained insignificant.

In further analyses, we examined whether outcomes change significantly across different socioeconomic subgroups. For this purpose, subgroups were stratified by sex (Table 3), age (Table 4), educational level (Table 5), and income groups (Table 6). However, no significant relationship between change in log income and the outcome measures of morbidity was found in any of these subgroups either (for example, physician-diagnosed diseases, among women: ß = −0.01, *p* = 0.89, among men: ß = −0.07, *p* = 0.34).

In addition, regression models were checked for multicollinearity (using the variance inflation factor). Nevertheless, we could not identify a collinearity problem (i.e., all variance inflation factors were lower than 2.98).

In summary, no significant association was found between log income change and any of the three outcome measures. Neither the use of a second income variable (log net household income) nor the examination of different socioeconomic subgroups resulted in a significant relationship between income and morbidity.

It should be noted that increases in morbidity were associated with increases in age or worsening self-rated health. These associations were consistent across all analyses. The other covariates were not consistently associated with the outcome measures.

## 4. Discussion

### 4.1. Main Findings

The aim of this study was to investigate the relationship between percentage change in income and self-reported diseases, physician-diagnosed diseases, and current symptoms. Therefore, longitudinal data from a large nationally representative survey of people in the second half of life in Germany were analyzed. Using fixed-effects regressions, no significant association between income and morbidity was found. Further analyses of specific socioeconomic groups within the total sample also showed no significant association over time. Increases in morbidity were significantly associated with increases in age and worsening self-rated health across all analyses.

### 4.2. Relation to Previous Research

Looking at previous literature, there are studies that have investigated the relationship between income and health and found a significant association. The vast majority of them used cross-sectional data (e.g., [45,46]). However, the use of longitudinal data has been found to be clearly superior to cross-sectional data for investigating a causal relationship [15]. Compared to cross-sectional studies, only a few longitudinal studies found significantly smaller associations between income and health [8]. In addition, many used the subjective health measure of self-rated health, although this is highly susceptible to attenuation bias [6]. While most longitudinal studies found a significantly positive association between income and self-rated health, some studies found a negative association [9,47]. Thus, the results of these studies are inconclusive. It should also be noted that the findings varied depending on the fact of whether fixed-effects regressions or, for example, random-effects regressions were used when longitudinal data were available [44]. On the other hand, there are very few studies that used longitudinal data and more objective health variables such as morbidity [14]. In the few studies that met these criteria, a slightly significant negative relationship between income and morbidity was found in the United States and China [18,48]. Initially, we assumed that a significant negative association would also be found in this study. We expected that higher income could bring a health advantage since additional services, for example, in preventive care, could be used at the doctor’s office [3]. Since better and healthier foods are more expensive than unhealthy ones, a higher income is also more likely to enable a healthy diet [1]. Moreover, the infrastructure of a residence tends to deteriorate as the standard of living decreases. In other words, poor residential areas often lack well-stocked supermarkets and facilities for physical activity and health care. In contrast, more expensive neighborhoods offer better access to such infrastructure [49]. However, this expectation was not fulfilled. Thus, contrary to the previous literature, the results of this study increasingly call into question the link between income change and health (in terms of morbidity).

### 4.3. Possible Explanatory Factors

The observed absence of a significant correlation could be explained, on the one hand, by using longitudinal data and fixed effects regression analyses and by controlling for many different confounding factors. These methods made it possible to test whether there is a causal relationship between income and morbidity. On the other hand, the comparable rather good social security and health care system in Germany could be an explanation for these results [50]. We assume that good access to health care is available to everyone in Germany, regardless of employment status and income. In the United States, for example, health insurance is often tied to jobs, and medical treatments must be paid for significantly more often by patients themselves [51]. Therefore, income probably plays a greater role in health in the United States. Moreover, Germany is a prosperous country with comparably little absolute poverty overall. This may also have contributed to why we did not identify a significant association between income change and morbidity.

We also assume that there are other possible influencing time-varying factors we could not control for because they were not included in the sample, which could also have led to insignificance. As an example, it is well known that the prevention of chronic diseases can be achieved mainly through individual health behavior and attitudes towards health [26,52]. Thus, the availability of physical activity and the implementation of healthy nutrition are important influencing factors. The knowledge of good health behavior (health literacy) is probably, in turn, strongly dependent on the level of education, which could thus have a strong impact on health. In addition, we assume that another explanatory factor could be the time available for individual health care. What is the point of having lots of money but hardly any time for doctor’s visits, exercise, or healthy nutrition?

In summary, based on data from a big nationally representative survey of Germans in the second half of life, this study raises increasing doubt on the relationship between income and health (in terms of morbidity).

As mentioned earlier, the health care system, the wealth of the country, individual health behavior, and time available for personal health care are possible explanatory factors for why we did not find a significant association between income change in percentage terms and morbidity.

### 4.4. Strengths and Limitations

This study is one of the few studies that have examined the relationship between income changes and morbidity over time, and it is the first one to use data from a German panel survey to investigate this issue. In the previous literature, there were many studies that used cross-sectional data but only a few that explored longitudinal data. We used longitudinal data in our study because this allowed us to control for any unobserved time-constant individual factors (both observed and unobserved), thus having greater explanatory power than with the use of cross-sectional data [15]. Our research was based on data from the German Ageing Survey, which is a large nationally representative survey with a balanced sample distribution of nearly 50% each of men and women. However, the sample, which includes only community-dwelling persons, excludes individuals from nursing facilities. Thus, future research is required to clarify the association between income and morbidity among individuals living in institutionalized settings. This is of particular relevance since the number of people in need of care may increase substantially in the coming decades due to the aging baby boomer generation [53].

To achieve the best possible comparability and low bias of the income variable, we used net equivalized household income and logarithmized it. Equivalent income has the advantage of better comparability between different household compositions (single household vs. family of four) [27]. By logarithmizing the variable, percentage changes in income can be mapped, which, in turn, creates better comparability between different income groups. It is important to note that the income data was based on self-reporting only and may, therefore, have led to a bias in the results. Indeed, with self-reported income data, there is always a risk of underestimation of actual income. On the other hand, there is no bias in the fixed-effects estimators if a possible underestimation of income remains constant over time [54]. Nevertheless, if possible, more objective sources such as income tax statistics should be preferred. However, this type of data is, most often, unavailable.

In order to get a comprehensive picture of morbidity, we used not only one but three different variables. For morbidity, there is a similar limitation (as described above) to income. Self-reported diseases and self-reported severity of diseases are particularly predisposed to bias due to subjective evaluation by participants. With the third variable, physician-diagnosed diseases, we added a more objective variable.

The German Ageing Survey has a rather low response rate. Nevertheless, studies have shown that the distribution of sociodemographic characteristics in the sample, such as marital status and household composition, is very similar to that in the German population. A possible selection effect, therefore, remains small [29,55].

Overall, it can be said that we reduced the unobserved heterogeneity by controlling for several potential confounders and used fixed-effects regressions to control for time-invariant factors. However, the causality of the relationship between income change and morbidity should be further explored. Thus, studies with experimental income shocks such as lottery winnings or unconditional income might provide a better basis to assess causality. In previous literature, there were two studies that showed contradictory results when using lottery winnings as income shocks. While the study from Great Britain found no significant association between income change and physical health [56], the study from Sweden found a negative association between income change and chronic diseases [57]. Consequently, further research using these income shocks is required to provide more clarity on the association between income and morbidity.

## 5. Conclusions

After analyzing data from four waves of the German Ageing Survey, we conclude that there is no significant association between income change and morbidity among older adults in Germany. No significant association with income was found for either self-reported diseases, physician-diagnosed diseases, or self-reported disease severity. Even when certain socioeconomic groups were considered, no significant correlation was found.

This means that the initial hypothesis that there is a significant relationship between income and morbidity over time could not be confirmed.

Instead, it may be useful to pay special attention to other factors, such as the health locus of control, which might contribute to morbidity [58]. In this context, another interesting factor, for example, based on intervention strategies, may be health literacy [59].

Accordingly, the long-assumed link between income and health should be increasingly questioned. However, there is still a lack of research using longitudinal data with the variable of morbidity and a large research gap in analyzing data from developing countries. Therefore, more research should be done in this regard, particularly using longitudinal data and morbidity as a variable for health. Since Germany is one of the wealthiest countries in the world, with a very good health and social security system, it is also essential to study the research topic in other countries that are less wealthy and have less developed health and social security systems. Income may play a greater role in access to health care—and perhaps health—in these countries.

It might also be interesting to compare individuals with income increases over time to those with income decreases over time in terms of morbidity. In this regard, more research should also be done.

Furthermore, it would be interesting to examine the association between income and health, specifically during the COVID-19 pandemic. Due to short-time work, job losses, the additional threat to health caused by the virus, and the lack of access to sports activities, the association may have changed.

## Figures and Tables

**Table 1:** Sample characteristics.

Variables	N (%)/Mean (SD); Range
Total sample (Observations)	13,027
Sex	
1. Male	6555 (50.3%)
2. Female	6472 (49.7%)
Age	62.4 (10.8); 40–95
Education level	
1. Low (ISCED 0–2)	915 (7.0%)
2. Medium (ISCED 3–4)	6829 (52.4%)
3. High (ISCED 5–6)	5282 (40.5%)
Family structure	
1. Married, living together with spouse	9314 (71.5%)
2. Married, living separated from spouse	203 (1.6%)
3. Divorced	1282 (9.8%)
4. Widowed	1336 (10.3%)
5. Single	892 (6.8%)
Employment status	
1. Working	4808 (36.9%)
2. Retired	6774 (52.0%)
3. Other (not employed)	1445 (11.1%)
Social class	
1. lower class	498 (3.8%)
2. lower middle class	2450 (18.8%)
3. middle class	3634 (27.9%)
4. upper middle class	4243 (32.6%)
5. upper class	2202 (16.9%)
Self-rated health (from 1 = very good to 5 = very bad)	2.5 (0.8); 1–5
Life satisfaction	3.8 (0.7); 1–5
Social network (number of important people in regular contact)	5.0 (2.8); 0–9
Loneliness	1.8 (0.5); 1–4
Physical functioning	83.8 (21.8); 0–100
Depressive symptoms	6.5 (6.1); 0–45
Net household equivalent income (in EUR)	1847.2 (1497.2); 1–65,000
Net household income (in EUR)	2859.1 (2317.7); 1–97,500
Number of self-reported diseases (11 common diseases)	2.4 (1.8); 0–11
Number of physicians diagnosed diseases (19 common diseases)	2.3 (1.7); 0–14
Self-reported severity of diseases (count score)	6.6 (5.7); 0–41

Notes: The education level was measured using the International Standard Classification of Education (ISCED) by UNESCO. The Satisfaction With Life Scale (SWLS) was used to quantify life satisfaction. To measure physical functioning, the subscale “Physical functioning” of the Short Form Health Survey (SF-36) was applied. The Center of Epidemiological Studies depression scale (CES-D) was used to quantify depressive symptoms. The 6-item De Jong Gierveld Loneliness Scale was used as a measurement instrument for overall, emotional, and social loneliness to quantify loneliness.

**Table 2:** Determinants of morbidity. Results of linear fixed-effects regressions.

Independent Variables	Self-Reported Diseases	Self-Reported Diseases	Physician Diagnosed Diseases	Physician Diagnosed Diseases	Self-Reported Severity of Diseases	Self-Reported Severity of Diseases
Log net household equivalent income	−0.03 (0.06)		−0.04 (0.05)		−0.16 (0.18)	
Log net household income		−0.05 (0.06)		−0.03 (0.05)		−0.11 (0.19)
Potential confounders	√	√	√	√	√	√
R^2^	0.05	0.05	0.10	0.10	0.09	0.09
Observations	13,027	13,040	13,193	13,207	13,193	13,207
Number of individuals	9810	9817	9905	9913	9905	9913

Notes: Potential confounders include age, self-rated health, life satisfaction, family structure, employment status, social network, loneliness, physical functioning, depressive symptoms, and social class. Unstandardized beta-coefficients are reported. Robust standard errors in parentheses. *** *p* < 0.001, ** *p* < 0.01, * *p* < 0.05, + *p* < 0.10; list-wise deletion was used to handle missing data.

**Table 3:** Determinants of morbidity. Results of linear fixed-effects regressions (stratified by sex).

-Sex-						
	Women			Men		
Independent Variable	Self-Reported Diseases	Physician Diagnosed Diseases	Self-Reported Severity of Diseases	Self-Reported Diseases	Physician Diagnosed Diseases	Self-Reported Severity of Diseases
Log net household equivalent income	−0.01 (0.08)	−0.01 (0.07)	0.00 (0.24)	−0.03 (0.09)	−0.07 (0.07)	−0.28 (0.25)
Potential confounders	√	√	√	√	√	√
R^2^	0.05	0.10	0.08	0.08	0.12	0.11
Observations	6472	6549	6549	6555	6644	6644
Number of individuals	4857	4896	4896	4953	5009	5009

Notes: Potential confounders include age, self-rated health, life satisfaction, family structure, employment status, social network, loneliness, physical functioning, depressive symptoms, and social class. Unstandardized beta-coefficients are reported. Robust standard errors in parentheses. *** *p* < 0.001, ** *p* < 0.01, * *p* < 0.05, + *p* < 0.10; list-wise deletion was used to handle missing data.

**Table 4:** Determinants of morbidity. Results of linear fixed-effects regressions (stratified by age).

-Age Group-						
	40–64 Years			65 and Older		
Independent Variable	Self-Reported Diseases	Physician Diagnosed Diseases	Self-Reported Severity of Diseases	Self-Reported Diseases	Physician Diagnosed Diseases	Self-Reported Severity of Diseases
Log net household equivalent income	−0.04 (0.07)	−0.08 (0.06)	−0.08 (0.21)	−0.00 (0.16)	−0.04 (0.14)	−0.03 (0.49)
Potential confounders	√	√	√	√	√	√
R^2^	0.07	0.13	0.10	0.06	0.09	0.10
Observations	7114	7192	7192	5913	6001	6001
Number of individuals	5391	5433	5433	5019	5088	5088

Notes: Potential confounders include age, self-rated health, life satisfaction, family structure, employment status, social network, loneliness, physical functioning, depressive symptoms, and social class. Unstandardized beta-coefficients are reported. Robust standard errors in parentheses. *** *p* < 0.001, ** *p* < 0.01, * *p* < 0.05, + *p* < 0.10; list-wise deletion was used to handle missing data.

**Table 5:** Determinants of morbidity. Results of linear fixed-effects regressions (stratified by education level).

-Education Level-									
	Low			Medium			High		
Independent Variable	Self-Reported Diseases	Physician Diagnosed Diseases	Self-Reported Severity of Diseases	Self-Reported Diseases	Physician Diagnosed Diseases	Self-Reported Severity of Diseases	Self-Reported Diseases	Physician Diagnosed Diseases	Self-Reported Severity of Diseases
Log net household equivalent income	−0.23 (0.30)	−0.02 (0.22)	−1.50 (1.00)	−0.03 (0.09)	−0.08 (0.08)	−0.14 (0.28)	−0.03 (0.08)	−0.02 (0.06)	−0.13 (0.24)
Potential confounders	√	√	√	√	√	√	√	√	√
R^2^	0.07	0.15	0.12	0.06	0.11	0.09	0.06	0.10	0.11
Observations	915	931	931	6829	6911	6911	5282	5350	5350
Number of individuals	751	762	762	5201	5249	5249	3857	3893	3893

Notes: Educational level was quantified using ISCED-97 classification. Potential confounders include age, self-rated health, life satisfaction, family structure, employment status, social network, loneliness, physical functioning, depressive symptoms, and social class. Unstandardized beta-coefficients are reported. Robust standard errors in parentheses. *** *p* < 0.001, ** *p* < 0.01, * *p* < 0.05, + *p* < 0.10; list-wise deletion was used to handle missing data.

**Table 6:** Determinants of morbidity. Results of linear fixed-effects regressions (stratified by income, median split).

-Income Groups (Median Split)-						
	Low (<Median)			High (>Median)		
Independent Variable	Self-Reported Diseases	Physician Diagnosed Diseases	Self-Reported Severity of Diseases	Self-Reported Diseases	Physician Diagnosed Diseases	Self-Reported Severity of Diseases
Log net household equivalent income	0.03 (0.10)	−0.10 (0.10)	−0.04 (0.34)	0.03 (0.11)	0.02 (0.08)	0.16 (0.28)
Potential confounders	√	√	√	√	√	√
R^2^	0.06	0.10	0.09	0.05	0.10	0.11
Observations	6681	6775	6775	6346	6418	6418
Number of individuals	5405	5465	5465	4979	5028	5028

Notes: Income groups were formed by determining median net equivalized household income. The groups below and above the median were considered. Potential confounders include age, self-rated health, life satisfaction, family structure, employment status, social network, loneliness, physical functioning, depressive symptoms, and social class. Unstandardized beta-coefficients are reported, robust standard errors in parentheses; *** *p* < 0.001, ** *p* < 0.01, * *p* < 0.05, + *p* < 0.10, list-wise deletion was used to handle missing data.

## Data Availability

The data used in this study are third-party data. The anonymized data sets of the DEAS (1996, 2002, 2008, 2011, 2014, 2017, and 2020) are available for secondary analysis. The data has been made available to scientists at universities and research institutes exclusively for scientific purposes. The use of data is subject to written data protection agreements. Microdata of the German Ageing Survey (DEAS) are available free of charge to scientific researchers for non-profitable purposes. The FDZ-DZA provides access and support to scholars interested in using DEAS for their research. However, for reasons of data protection, signing a data distribution contract is required before data can be obtained. For further information on the data distribution contract, please see https://www.dza.de/en/research/fdz/access-to-data/formular-deas-en-english (accessed on 13 November 2021).

## Supplementary Materials

The following are available online at https://www.mdpi.com/article/10.3390/ijerph182312365/s1, Table S1: The detailed lists of self-reported and physician-diagnosed diseases analyzed in the German Ageing Survey, Table S2: Overview of the independent and dependent variables, Table S3: Determinants of morbidity. Results of linear fixed-effects regressions, including coefficients of potential confounders, Table S4: Determinants of morbidity. Results of linear fixed-effects regressions, adjusting for sociodemographic factors, Table S5: Determinants of morbidity. Results of linear fixed-effects regressions, adjusting for sociodemographic and psychosocial factors.
