# Supplementary material for: The Relationship between Income and Morbidity—Longitudinal Findings from the German Ageing Survey
Source: Int J Environ Res Public Health. 2021 Nov 24;18(23):12365. doi: 10.3390/ijerph182312365 (PMC8656553; doi:10.3390/ijerph182312365)
Supplement: Supplementary file 1 [file ijerph-18-12365-s001.zip › ijerph-1444313-supplementary.pdf]

Supplementary Materials

Supplementary Table S1: The detail lists of self-reported and physician diagnosed diseases analyzed in the German Ageing Survey

Self-reported diseases	Physician diagnosed diseases
1. Cardiac and circulatory disorders	1. High cholesterol
	2. High blood pressure
	3. Heart attack, angina pectoris
	4. Cardiac insufficiency including coronary artery diseases
	5. Stroke
2. Bad circulation	6. Circulatory disorders in the brain
	7. Circulatory disorders in the legs
3. Joint, bone, spinal or back problems	8. Joint degeneration (arthrosis) of the hips, knees, or spine
	9. Osteoporosis
	10. Inflammatory joint or spinal diseases (arthritis or rheumatoid arthritis)
4. Respiratory problems, asthma, shortness of breath	11. Chronic pulmonary diseases (e.g., chronic bronchitis, pulmonary emphysema)
5. Stomach and intestinal problems	12. Stomach ulcer, intestinal ulcer
6. Cancer	13. Cancer, malignant tumor (including leukemia)
7. Diabetes	14. Diabetes, high blood sugar levels
8. Gall bladder, liver, or kidney problems	
9. Bladder problems	15. Incontinence
10. Eye problems, vision impairment	16. Glaucoma or macular degeneration
11. Ear problems, hearing problems	
	17. Parkinson`s disease
	18. Mental illness (e.g., panic attacks, depression, psychosis)
	19. Other chronic disease or health condition

Supplementary Table S2: Overview of the dependent and independent variables

Dependent variables	Morbidity	Self-reported diseases: Count score based on the number of self-reported diseases (list of 11 common diseases)
		Physician diagnosed diseases: Count score based on the number of physicians diagnosed diseases (list of 19 common diseases)
		Self-reported severity of diseases: Count score based on a scale from 1(none) to 4(severe) for current complaints of each self-reported disease
Independent variables	Income	Log net equivalent household income (weighted according to the modified OECD equivalent scale)
		Log net household income

Supplementary Table S3: Determinants of morbidity. Results of linear fixed effects regressions including the coefficients of potential confounders.

Independent variables	Self-reported diseases	Self-reported diseases		Physician diagnosed diseases	Physician diagnosed diseases		Self-reported severity of diseases	Self-reported severity of diseases
Log net household equivalent	-0.03			-0.04			-0.16	
income	(0.06)			(0.05)			(0.18)	
Log net household income		-0.05			-0.03			-0.11
		(0.06)			(0.05)			(0.19)
Age	0.05***	0.05***		0.06***	0.06***		0.17***	0.17***
	(0.01)	(0.01)		(0.01)	(0.01)		(0.02)	(0.02)
Self-rated health	0.18***	0.18***		0.14***	0.14***		0.75***	0.75***
	(0.03)	(0.03)		(0.03)	(0.03)		(0.11)	(0.11)
Life satisfaction	-0.14**	-0.14**		-0.05	-0.05		-0.59***	-0.59***
	(0.05)	(0.05)		(0.04)	(0.04)		(0.15)	(0.15)
Family structure (in reference to “married, living with spouse”)								
Married, living separate	0.06	0.04		0.13	0.12		-0.46	-0.48
from spouse	(0.19)	(0.19)		(0.17)	(0.17)		(1.06)	(1.06)

Divorced	0.19	0.17		-0.28*	-0.28*		-0.14	-0.17
	(0.20)	(0.20)		(0.11)	(0.11)		(0.60)	(0.60)
Widowed	0.45+	0.44+		0.20	0.19		1.31*	1.27*
	(0.23)	(0.24)		(0.15)	(0.16)		(0.59)	(0.59)
Single	0.70*	0.69*		0.06	0.06		1.31	1.31
	(0.33)	(0.33)		(0.21)	(0.21)		(0.96)	(0.96)
Employment status (in reference to “employed”)								
Retired	-0.05	-0.06		-0.06	-0.07		-0.19	-0.19
	(0.07)	(0.07)		(0.06)	(0.06)		(0.20)	(0.20)
Other: not employed	0.02	0.03		0.07	0.07		-0.05	-0.01
	(0.07)	(0.07)		(0.06)	(0.06)		(0.20)	(0.20)
Social network	0.01	0.01		0.03***	0.03***		0.06*	0.06*
	(0.01)	(0.01)		(0.01)	(0.01)		(0.02)	(0.02)
Loneliness	0.20***	0.21***		0.07	0.05		0.64***	0.65***
	(0.06)	(0.06)		(0.05)	(0.05)		(0.18)	(0.18)

Physical functioning	-0.00	-0.00		-0.01***	-0.01***		-0.02**	-0.02**
	(0.00)	(0.00)		(0.00)	(0.00)		(0.01)	(0.01)
Depressive symptoms	0.00	0.00		0.02***	0.02***		0.03*	0.03*
	(0.00)	(0.00)		(0.00)	(0.00)		(0.01)	(0.01)
Social class (in reference to “lower class”)								
Lower middle class	0.17	0.17		0.32	0.32+		0.35	0.38
	(0.20)	(0.20)		(0.20)	(0.20)		(0.60)	(0.60)
Middle class	0.05	0.04		0.24	0.25		0.10	0.08
	(0.20)	(0.20)		(0.20)	(0.20)		(0.61)	(0.61)
Upper middle class	0.05	0.04		0.27	0.28		0.10	0.09
	(0.21)	(0.21)		(0.20)	(0.20)		(0.62)	(0.62)
Upper class	0.02	0.01		0.30	0.30		0.32	0.31
	(0.23)	(0.23)		(0.20)	(0.20)		(0.66)	(0.66)
R^2	0.05	0.05		0.10	0.10		0.09	0.09

Observations	13,027	13,040		13,193	13,207		13,193	13,207
Number of individuals	9,810	9,817		9,905	9,913		9,905	9,913

Notes: Unstandardized beta-coefficients are reported, Robust standard errors in parentheses; *** p<0.001, ** p<0.01, * p<0.05, + p<0.10, list-wise deletion was used to handle missing data.

Supplementary Table S4: Determinants of morbidity. Results of linear fixed effects regressions adjusting for sociodemographic factors

Independent variables	Self-reported diseases	Self-reported diseases	Physician diagnosed diseases	Physician diagnosed diseases	Self- reported severity of diseases	Self- reported severity of diseases
Log net household equivalent income	-0.08<br>(0.06)		-0.01<br>(0.05)		-0.18<br>(0.18)	
Log net household income		-0.11+<br>(0.06)		0.00<br>(0.05)		-0.03<br>(0.19)
Potential sociodemographic confounders	✓	✓	✓	✓	✓	✓
R^2	0.02	0.02	0.04	0.04	0.02	0.02

Observations	13,486	13,501	17,393	17,421	17,393	17,421
Number of individuals	10,099	10,107	12,828	12,843	12,828	12,843

Notes: Potential sociodemographic confounders include age, employment status, family structure, social class;
Unstandardized beta-coefficients are reported; Robust standard errors in parentheses; *** p<0.001, ** p<0.01, * p<0.05, + p<0.10, list-wise deletion was used to handle missing data.

Supplementary Table S5: Determinants of morbidity. Results of linear fixed effects regressions adjusting for sociodemographic and psychosocial factors

Independent variables	Self-reported diseases	Self-reported diseases	Physician diagnosed diseases	Physician diagnosed diseases	Self-reported severity of diseases	Self-reported severity of diseases
Log net household equivalent income	-0.06<br>(0.06)		-0.05<br>(0.05)		-0.28<br>(0.18)	
Log net household income		-0.08<br>(0.06)		-0.04<br>(0.05)		-0.27<br>(0.19)
Potential sociodemographic and psychosocial confounders	✓	✓	✓	✓	✓	✓
R^2	0.04	0.04	0.06	0.06	0.05	0.05
Observations	13,269	13,284	13,441	13,457	13,441	13,457

Number of individuals	9,966	9,974		10,064	10,073		10,064	10,073

Notes: Potential sociodemographic and psychosocial confounder include age, employment status, family structure, social class, life satisfaction, loneliness, social network; Unstandardized beta-coefficients are reported; Robust standard errors in parentheses; *** $p<0.001$, ** $p<0.01$, * $p<0.05$, + $p<0.10$
